# Forming Facial Expressions Influences Assessment of Others' Dominance but Not Trustworthiness

**DOI:** 10.3389/fpsyg.2017.02097

**Published:** 2017-12-01

**Authors:** Yoshiyuki Ueda, Kie Nagoya, Sakiko Yoshikawa, Michio Nomura

**Affiliations:** ^1^Kokoro Research Center, Kyoto University, Kyoto, Japan; ^2^Faculty of Education, Kyoto University, Kyoto, Japan; ^3^Graduate School of Education, Kyoto University, Kyoto, Japan

**Keywords:** facial feedback, physiological state, happy face, disgusted face, dominance, trustworthiness, personal traits

## Abstract

Forming specific facial expressions influences emotions and perception. Bearing this in mind, studies should be reconsidered in which observers expressing neutral emotions inferred personal traits from the facial expressions of others. In the present study, participants were asked to make happy, neutral, and disgusted facial expressions: for “happy,” they held a wooden chopstick in their molars to form a smile; for “neutral,” they clasped the chopstick between their lips, making no expression; for “disgusted,” they put the chopstick between their upper lip and nose and knit their brows in a scowl. However, they were not asked to intentionally change their emotional state. Observers judged happy expression images as more trustworthy, competent, warm, friendly, and distinctive than disgusted expression images, regardless of the observers' own facial expression. Observers judged disgusted expression images as more dominant than happy expression images. However, observers expressing disgust overestimated dominance in observed disgusted expression images and underestimated dominance in happy expression images. In contrast, observers with happy facial forms attenuated dominance for disgusted expression images. These results suggest that dominance inferred from facial expressions is unstable and influenced by not only the observed facial expression, but also the observers' own physiological states.

## Introduction

Studies have established that observers' physiological (somatic) states influence their emotions, cognitions, and perceptions (e.g., Zajonc, 1980). This is called *embodied cognition*, indicating that cognitive processes might be shaped by both the brain and the body, including the motor system and the perceptual system. For example, variations in facial muscles (associated with facial expressions) lead to changes in emotions and impressions of objects (Strack et al., 1988; Soussignan, 2002; Wiswede et al., 2009; Davey et al., 2013). Through increasing in autonomic arousal and self-reported emotional experiences, in general, forming smiling faces makes expressers feel more positive, and forming frowning faces makes them feel more negative (Davis et al., 2009; Lee et al., 2013).

Given this, changes in perceivers' physiological states should be considered in the context of social perception. This point of view is important to reveal the cognitive mechanisms that apply to actual social interaction in which perceivers' states are not always neutral. However, studies in this field have investigated the perception of personal traits via responses to facial expressions presented to observers (e.g., Eastwood et al., 2001; Öhman et al., 2001; Leppännen and Hietanen, 2004; Ito et al., 2010); recently, some studies have investigated the effect of perceivers' age and disorders (Di Domenico et al., 2015; Altamura et al., 2016), although few have investigated the effect of the perceivers' temporal physiological states. Hence, to fill this gap and reveal the influence of perceivers' physiological states on social perception, we investigated the effect of perceivers' facial expressions on their perception of the personal traits of others.

To engage in appropriate behaviors in human society, humans have to infer others' personal traits as quickly and accurately as possible. This study specifically focused on the ability to perceive trustworthiness and dominance, which are particularly important in social interactions. Trustworthiness is a sign of potential cooperation, and dominance is a sign of a hierarchy for social behaviors (Zebrowitz et al., 2015; see Todorov et al., 2008, for a review). If someone is not trustworthy, we should not provide that person with information that might result in betrayal. Likewise, if an individual is dominant over us, we should be careful not to offend that person. Facial expression is one of the nonverbal signals that can convey these traits (Hall et al., 2005; Todorov et al., 2008; Ueda and Yoshikawa, 2017).

To address a perceiver's state, we used a *facial feedback* method that has been suggested as a valid manipulations (Strack et al., 1988; Soussignan, 2002; Wiswede et al., 2009; Davey et al., 2013; Meeten et al., 2015; Kaiser and Davey, 2017). In this method, participants are often asked to make a specific facial form using a pen (Wiswede et al., 2009): For example, participants are frequently asked to bite a pen with their teeth, which causes their lip-corners to raise. The resulting face formed looks like a smiling face, indicating that the facial musculature has contracted as it would when people intentionally smile. Previous studies have showed that participants forming such faces report more positive experiences when presented with pleasant scenes and humorous cartoons (Strack et al., 1988; Soussignan, 2002). Additionally, participants who were asked to knit their brows, forming disgusted or scowling faces, were more likely to interpret homophones that had threatening and neutral meanings as threats (Davey et al., 2013). These results suggest that participants' physiological states of facial expression can influence their cognition. Importantly, in this method, participants were not asked to intentionally change their emotions or cognitive strategy.

Previous studies have showed that compared with expressions of anger and disgust, people with a happy expression are perceived as less dominant (Keating et al., 1981; Hall et al., 2005; Todorov et al., 2008; Ueda and Yoshikawa, 2017). Therefore, we expected that (a) observed individuals who displayed a happy expression would be regarded as less dominant. However, dominance is judged not only absolutely but also relatively: Since dominance is regarded as the power that enables people to get their own way over other people, observed individual dominance might be judged less intensely if observers are more dominant. This is supported by the results of previous studies showing that, when facing a person who expresses disgust, maintaining a happy expression engenders positive and confident moods, which are associated with the inference of dominance, and leads to a more dominant impression (Tracy et al., 2013; Ueda and Yoshikawa, 2017). Then, we hypothesized that (b) the dominance of individuals would be judged as relatively low when observers formed happy expressions compared with when they formed disgusted expressions.

Trustworthiness connotes a partner's cooperative intentions. Therefore, regardless of observers' facial expressions, a partner's intentions might be invariant. Considering the previous studies showing that a happy expression is associated with perceived trustworthiness (Oosterhof and Todorov, 2009; Caulfield et al., 2014), our final hypothesis was that (c) perceived trustworthiness would be higher when observed individuals showed happy expressions and would not change regardless of observers' expressions.

In Experiment 1, we asked participants to rate the trustworthiness and dominance of the person shown on the screen. During the viewing, the observers were asked to make happy, neutral, and disgusted facial expressions: for “happy,” they held a wooden chopstick in their molars to form a smile; for “neutral,” they clasped the chopstick between their lips, making no expression; for “disgusted,” they put the chopstick between their upper lip and nose and knit their brows in a scowl. However, they were not asked to intentionally change their emotional state and were not explicitly instructed to form these expressions. In Experiment 2, to expand the results of Experiment 1, we asked the participants to rate other properties of the person on the screen in addition to trustworthiness and dominance.

## Experiment 1

### Methods

#### Participants

Sixty participants (32 females and 28 males, average age 20.8 ± 2.32 years) attending Kyoto University participated; two were subsequently excluded due to imperfection of data collection. All reported normal or corrected-to-normal vision. Each was paid JPN¥500 for their participation in the half-hour experiment. Sample size was determined on the basis of a power analysis using middle effect size (*f* = 0.25) and power (0.90). The internal review board of Kyoto University approved the procedures.

#### Apparatus

The stimuli were presented on a 32-inch monitor with a resolution of 1,360 × 768 pixels, at a viewing distance of 130 cm. Stimulus presentation was controlled by MATLAB (MathWorks) with the Psychophysics Toolbox version 3.0.8 (Brainard, 1997; Pelli, 1997; http://psychtoolbox.org/). The position of each observer participant's head was not fixed, although he or she was asked to minimize head movements. Each participant's facial expressions were monitored by a camcorder.

#### Stimuli

Twenty-four female faces^1^ were chosen from the Kokoro Research Center (KRC) facial expression database. For each participant, half of the faces were randomly chosen to show a happy expression and the others were presented with a disgusted expression. Twenty-seven participants, including 13 females, were asked to rate each photo in terms of the emotions “happiness” and “disgust” on a 7-point scale (1 = *very weak*, 7 = *very intense*). The mean intensity of happiness for faces used in this experiment were as follows: happy expression = 4.76 (*SD* 0.61) and disgusted expression = 1.14 (*SD* 0.16). On the other side, the mean intensity of disgust were as follows: happy expression = 1.42 (*SD* 0.11), disgusted expression = 4.78 (*SD* 0.40). The visual angle subtended by each face was 17.1° tall × 12.3° width.

#### Procedure

**Figure 1:**
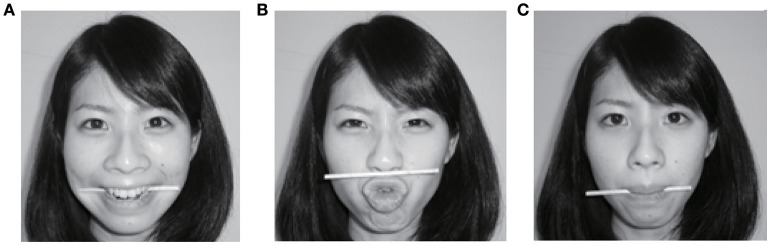
Examples of facial expressions formed using a wooden chopstick. The images correspond to **(A)** happy, **(B)** disgusted, and **(C)** neutral expressions.

Initially, the participants were randomly assigned to one of three facial expression conditions and were asked to make a specific facial form by manipulating a wooden chopstick (see Figure 1). Following Wiswede et al.'s (2009) procedures and Ohta et al.'s (2005) facial expression analysis with Japanese, in the happy condition, participants were instructed to bite the chopstick with their molars, forming a smile. In the disgusted condition, the participants were asked to put the chopstick between their upper lip and nose and to knit their brows in a scowl, forming a look of disgust. In the control or neutral condition, the participants were asked to clasp the chopstick between their lips, straining the muscles around their lips to avoid making any specific facial expressions. In each condition, participants were asked to make the facial form using the chopstick and were not asked to make a specific facial expression or to feel a specific emotion.

For each trial, one face was shown at the center of the monitor for 3 s, followed by a blank display for 1 s. Subsequently, participants were asked to rate how trustworthy the presented person was, using a 7-point Likert scale, which was presented for 2 s. After a blank display of 1 s, participants were asked to rate the dominance of the person depicted using a 7-point Likert scale within 2 s. Participants were asked to complete their rating before each scale display disappeared. Ratings could be changed freely while a questionnaire was displayed.

Participants completed 24 trials with a 30-s break after every sixth trial. There were 12 repetitions of each facial expression condition, which took place in random order.

### Results

**Figure 2:**
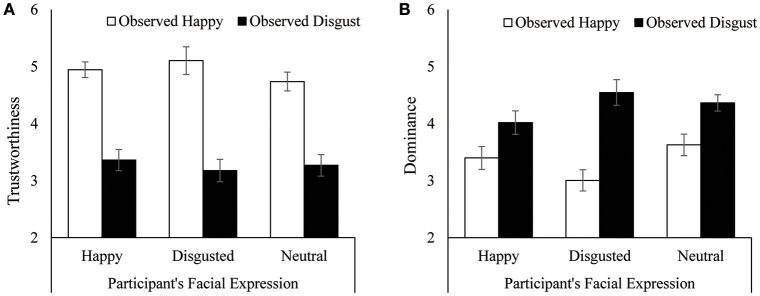
Mean ratings of observed facial expressions when the observer formed a specific facial expression in Experiment 1. **(A)** Trustworthiness. **(B)** Dominance. Error bars indicate standard error.

The average ratings of trustworthiness and dominance are shown in Figure 2. A 3 (participant's facial expression) × 2 (depicted facial expression) ANOVA on trustworthiness showed that individuals depicted with happy faces were judged to be more trustworthy than those who depicted disgust: *F*_(1, 55)_ = 139.17, *p* < 0.0001, η_p_^2^ = 0.72. However, ratings of trustworthiness were not influenced by the participant's facial expression nor by the interaction between the participant's facial expression and the depicted facial expression, both *F*s < 1. This result supported the third hypothesis.

For dominance judgments, a 3 × 2 ANOVA showed a significant main effect of depicted facial expression, *F*_(1, 55)_ = 35.19, *p* < 0.0001, η_p_^2^ = 0.39, indicating that individuals displaying disgusted faces were regarded as more dominant than those with happy expressions. This result supported the first hypothesis. Moreover, an interaction between the participant's facial expression and the depicted facial expression was also significant, *F*_(2, 55)_ = 3.06, *p* = 0.05, η_p_^2^ = 0.10. Follow-up analyses showed that although dominance judgments for depictions of disgust were rated higher than those of happiness, the differences between them were smaller when participants formed a happy expression, *F*_(1, 19)_ = 4.47, *p* = 0.05, η_p_^2^ = 0.19, than a disgusted expression, *F*_(1, 17)_ = 23.94, *p* = 0.0001, η_p_^2^ = 0.58, or a neutral expression, *F*_(1, 19)_ = 9.68, *p* = 0.006, η_p_^2^ = 0.34. On the other side, simple main effects of participant's facial appearance were not significant, *F*_(2, 55)_ = 2.60, *p* = 0.08, η_p_^2^ = 0.09 for depictions of happy, and *F*_(2, 55)_ = 1.93, *p* = 0.15, η_p_^2^ = 0.07 for depictions of disgusted. This did not support the second hypothesis: Instead, forming a happy expression decreased the difference of dominance assessments between depictions of happy and disgusted, whereas forming a disgusted expression increased difference of dominance judgments between them.

## Experiment 2

Experiment 1 showed that the effect of an observer's facial appearance on dominance was valid but not for gauging trustworthiness. To expand the results of Experiment 1 to investigate the effect of facial appearance on other social cognitions, we asked participants to rate competence, warmth, and friendliness of people in the same images used for rating trustworthiness and dominance. These personal traits correlated with trustworthiness of the person in the literature (Fiske et al., 2007; Todorov et al., 2008). Therefore, we again investigated whether trustworthiness was actually not influenced by an observer's facial expression using these personal traits. Based on the results of Experiment 1, we expected that perceptions of these personal traits may not be influenced by the participants' facial expressions. Moreover, we again investigated whether dominance judgment was influenced by observer's facial expressions. It was expected that we might get the same results as Experiment 1. In addition, we investigated the influence of the participants' facial appearance on perceived physical features separate from personal traits that corresponded to either happy or angry expressions. In this rating, participants were also asked to judge perceived facial distinctiveness. In this experiment, to detect subtle differences between conditions, we asked participants to rate them using a 9-point Likert scale within 3 s (1 s longer than in Experiment 1).

### Methods

#### Participants

Eighty-seven participants (44 females, average age 21.1 ± 1.71 years), who were different from the participants in Experiment 1, attending Kyoto University participated; eight were subsequently excluded because they could not respond within the required response period for more than 15% of responses, and one was excluded because she could not make the manipulated facial forms. All reported normal or corrected-to-normal vision. Each was paid JPN¥500 for their participation in the half-hour experiment. Sample size was determined on the basis of a power analysis using effect size (*f* = 0.30) and power (0.997) of Experiment 1. The internal review board of Kyoto University approved the procedures.

#### Apparatus and stimuli

All were the same as Experiment 1.

#### Procedure

The participants were randomly assigned to one of three facial expression conditions and the instructions for making facial forms were the same as Experiment 1.

For each trial, one face was shown at the center of the monitor for 3 s, followed by a blank display for 1 s. Subsequently, participants were asked to rate trustworthiness, dominance, competence, warmth, friendliness, and distinctiveness of the presented person using a 9-point Likert scale. Participants were instructed that a distinctive face was one that was easily recognized (this was the same procedure followed by Baudouin and Gallay, 2006). The order of the questionnaires was fixed, and participants had to complete their ratings within 3 s for each questionnaire. Between trials, a blank display of 1 s was presented.

### Results

**Figure 3:**
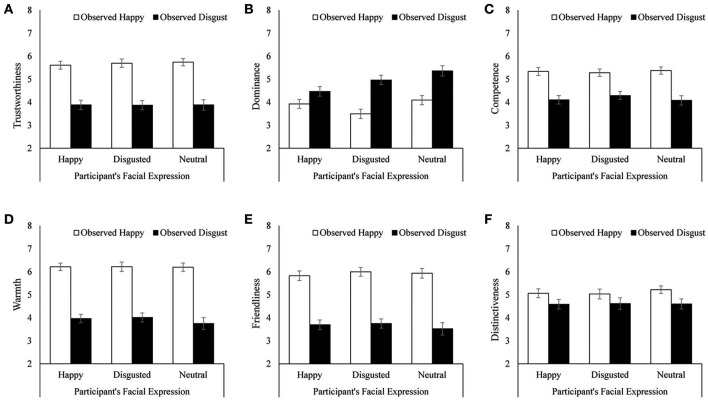
Mean ratings of observed facial expressions when the observer formed a specific facial expression in Experiment 2. **(A)** Trustworthiness. **(B)** Dominance. **(C)** Competence. **(D)** Warmth. **(E)** Friendliness. **(F)** Distinctiveness. Error bars indicate standard error.

The average ratings are shown in Figure 3. For dominance judgments, a 3 (participant's facial appearance) × 2 (depicted facial expression) ANOVA showed significant main effects of participant's facial appearance, *F*_(2, 75)_ = 3.67, *p* = 0.03, η_p_^2^ = 0.09, and depicted facial expression, *F*_(1, 75)_ = 48.84, *p* < 0.0001, η_p_^2^ = 0.39, indicating that a happy or disgusted expression decreased dominance judgments and that individuals displaying disgusted faces were regarded as more dominant. The latter results were consistent with those in Experiment 1 and again supported the first hypothesis. Moreover, an interaction between the participants' facial expression and the depicted facial expression was also significant, *F*_(2, 75)_ = 3.21, *p* = 0.05, η_p_^2^ = 0.08. Follow-up analyses showed that although dominance judgments for depictions of disgust were rated higher than those of happiness, the differences between them were smaller when participants formed a happy expression, *F*_(1, 25)_ = 5.53, *p* = 0.03, η_p_^2^ = 0.18, than a disgusted expression, *F*_(1, 25)_ = 29.45, *p* < 0.0001, η_p_^2^ = 0.54, or a neutral expression, *F*_(1, 25)_ = 17.19, *p* = 0.0003, η_p_^2^ = 0.41. These results were consistent with those of Experiment 1: Forming a happy expression decreased the difference in dominance assessments between depictions of happy and disgusted, whereas forming a disgusted expression increased the difference in dominance judgments between happy and disgusted. On the other side, a simple main effects of participant's facial appearance was significant for depictions of disgust, *F*_(2, 75)_ = 4.44, *p* = 0.02, η_p_^2^ = 0.11. Multiple comparisons showed that dominance of individuals showing disgust was rated lower when participants expressed a happy expression than when they expressed a disgusted expression (adjusted *p* = 0.01). However, a simple main effects of participant's facial appearance was not significant for depictions of happy, *F*_(2, 75)_ = 2.38, *p* = 0.10, η_p_^2^ = 0.06.

For other personal traits, 3 × 2 ANOVAs showed that happy faces were judged to be more trustworthy, competent, warm, friendly, and distinctive than disgust: trustworthiness, *F*_(1, 75)_ = 125.71, *p* < 0.0001, η_p_^2^ = 0.63; competence, *F*_(1, 75)_ = 68.43, *p* < 0.0001, η_p_^2^ = 0.48; warmth, *F*_(1, 75)_ = 165.52, *p* < 0.0001, η_p_^2^ = 0.69; friendliness, *F*_(1, 75)_ = 141.61, *p* < 0.0001, η_p_^2^ = 0.66; distinctiveness, *F*_(1, 75)_ = 20.20, *p* < 0.0001, η_p_^2^ = 0.21. However, these ratings were not influenced by the participants' facial expressions nor by the interaction between participants' and the depicted facial expressions, *F*s < 1, indicating that they were consistent with the results of trustworthiness in Experiment 1, and thus supported the third hypothesis.

## Discussion

Throughout two experiments, observed individuals who displayed a happy expression would be regarded as less dominant than who displayed a disgusted expression, supporting the first hypothesis and the result of previous studies (Keating et al., 1981; Hall et al., 2005; Todorov et al., 2008; Ueda and Yoshikawa, 2017). Furthermore, the results showed that judgments of dominance were influenced by not only observed facial expression but also the perceivers' facial expressions. Forming a happy expression decreased the difference in dominance assessments between depictions of happy and disgusted, whereas forming a disgusted expression increased the difference of dominance judgments between them. This was not congruent with the second hypothesis: the results suggest that the impact of the participants' expression was not the same for happy and disgusted faces. In contrast, judgments of trustworthiness, competence, warmth, friendliness, and distinctiveness were mainly influenced by observed facial expressions, but not by the perceivers' facial expressions. This supported the third hypothesis. If an individual appears happy, this signals cooperation; therefore, observers do not discount the expression, regardless of their own emotional state.

Follow-up analyses and multiple comparisons in Experiment 2 showed that differences of dominance assessments depending on participants' facial expressions were due to attenuation of perceived dominance for disgusted expression images while forming a smiling face. These analyses were not significant in Experiment 1, although interaction between the participants' facial appearance and their depicted facial expressions was significant. Therefore, the facial feedback effect might not only be attenuation of perceived dominance of disgusted faces while forming happy expressions. In addition, perceived dominance of disgusted faces might be reinforced and, at the same time, that of happy faces might be attenuated while forming disgusted expressions.

A scowling facial expression with knitted eyebrows suggests aggression and alludes to higher physical strength and perhaps greater levels of testosterone, whereas smiling suggests lower levels of these attributes (Dabbs, 1997; Kraus and Chen, 2013). Furthermore, some studies have showed that sympathetic activations such as heart rate and skin conductance are modulated by facial feedback techniques (Kraft and Pressman, 2012; Lee et al., 2013). Different modes of cognition based on these physiological states engendered by observer's facial expression feedback may explain why the results in this study did not fit the second hypothesis. The results indicate that forming a disgusted expression can lead not only to underestimating the dominance of a happy person, but also to overestimating the dominance of a disgusted person, suggesting that forming a disgusted facial expression may foster competitiveness. Conversely, forming a happy facial expression may foster cooperation and composure. Indeed, recent research has demonstrated that positive expressions are associated with cooperative thinking, whereas negative emotional expressions are associated with less cooperative thinking (Van Doorn et al., 2012). A pronounced smile formed by clenching a chopstick between the teeth, rather than forming a faint smile, may help maintain this mode of cognition. Since dominance requires the comparison of two people, these cognitive modes lead to different evaluations of an observed individual.

Moreover, this two-state model applies to trustworthiness. Viewing someone who is displaying a happy expression signals a cooperative mode, and display of a disgusted expression signals a competitive mode. Therefore, observers trust individuals who are expressing happiness, regardless of the observer's own facial expression.

Perceived dominance was lessened when participants formed a happy or disgusted expression—more so than when they formed a neutral expression—in Experiment 2, which may be due to the artifact of facial manipulation. Recently, Ahmed (2017) showed that facial expression categorization was impaired when observers' cognitive loads were high, suggesting that task demands change how we see presented faces. Since forming a neutral appearance strains only the muscles around the lips in conscious efforts to avoid making any specific facial expressions, this is relatively easier than forming happy or disgusted expressions. In Experiment 1, participants judged only trustworthiness and dominance; therefore the task demand was lower than in Experiment 2, where participant observers were asked to rate some personal traits simultaneously. Hence, this artifact might be less of a factor in Experiment 1.

In summary, observers' facial expressions did not affect their perceptions of the trustworthiness, competence, warmth, friendliness, and distinctiveness of other individuals, suggesting that assessing whether individuals will cooperate with the observers is independent of the observers' expressions. In contrast, perceived dominance is situationally dependent. Variations in social perception associated with the perceivers' physiological (somatic) states supports the notion proposed by James (1884) and Zajonc (1980) that affective judgments occur as a result of physiological reactions.

## Ethics statement

This study was carried out in accordance with the recommendations of the internal human ethics review board of Kyoto University with written informed consent from all subjects. All subjects gave written informed consent in accordance with the Declaration of Helsinki. The protocol was approved by the internal human ethics review board of Kyoto University.

## Author contributions

All authors developed the study concept and contributed to the study design. Data collection and analyses was mainly done by YU and KN. All authors drafted the paper and revised it. All authors approved the final version of the paper for submission.

### Conflict of interest statement

The authors declare that the research was conducted in the absence of any commercial or financial relationships that could be construed as a potential conflict of interest.

## Footnotes

^1^In our previous study, we got the same tendency of assessment of personality traits with both male faces and female faces (Ueda and Yoshikawa, 2017). Therefore, in this study, we used only female faces.
